# A comparison of human chorionic gonadotropin and luteinizing hormone releasing hormone on the induction of spermiation and amplexus in the American toad (*Anaxyrus americanus*)

**DOI:** 10.1186/1477-7827-10-59

**Published:** 2012-08-20

**Authors:** Andrew J Kouba, Javier delBarco-Trillo, Carrie K Vance, Callie Milam, Meghan Carr

**Affiliations:** 1 Conservation and Research Department, Memphis Zoo, 2000 Prentiss Place, Memphis, TN, 38112, USA; 2 University of Memphis, Biology Department, Ellington Hall, Memphis, TN, 38152, USA; 3 Biochemistry and Molecular Biology Department, Mississippi State University, Mississippi State, MS, 39759, USA; 4 Museo Nacional de Ciencias Naturales - CSIC, José Gutiérrez Abascal 2, Madrid, 28006, Spain

**Keywords:** Amphibian, Behavior, Hormones, Sperm, Toad

## Abstract

**Background:**

Captive breeding programs for endangered amphibian species often utilize exogenous hormones for species that are difficult to breed. The purpose of our study was to compare the efficacy of two different hormones at various concentrations on sperm production, quantity and quality over time in order to optimize assisted breeding.

**Methods:**

Male American toads (*Anaxyrus americanus*) were divided into three separate treatment groups, with animals in each group rotated through different concentrations of luteinizing hormone releasing hormone analog (LHRH; 0.1, 1.0, 4.0 and 32 micrograms/toad), human chorionic gonadotropin (hCG; 50, 100, 200, and 300 IU), or the control over 24 hours. We evaluated the number of males that respond by producing spermic urine, the sperm concentration, percent motility, and quality of forward progression. We also evaluated the effects of hCG and LHRH on reproductive behavior as assessed by amplexus. Data were analyzed using the Generalized Estimating Equations incorporating repeated measures over time and including the main effects of treatment and time, and the treatment by time interaction.

**Results:**

The hormone hCG was significantly more effective at stimulating spermiation in male *Anaxyrus americanus* than LHRH and showed a dose-dependent response in the number of animals producing sperm. At the most effective hCG dose (300 IU), 100% of the male toads produced sperm, compared to only 35% for the best LHRH dose tested (4.0 micrograms). In addition to having a greater number of responders (P < 0.05), the 300 IU hCG treatment group had a much higher average sperm concentration (P < 0.05) than the treatment group receiving 4.0 micrograms LHRH. In contrast, these two treatments did not result in significant differences in sperm motility or quality of forward progressive motility. However, more males went into amplexus when treated with LHRH vs. hCG (90% vs. 75%) by nine hours post-administration.

**Conclusion:**

There is a clear dichotomy between the two hormones’ physiological responses on gamete production and stimulation of amplexus. Understanding how these two hormones influence physiology and reproductive behaviors in amphibians will have direct bearing on establishing similar breeding protocols for endangered species.

## Background

The worldwide decline in amphibians has resulted in the establishment of numerous captive breeding assurance colonies by zoological, aquaria and governmental organizations. These assurance colonies are meant to serve as Arks that retain some portion of the populations’ biological diversity should a species go extinct in the wild. The goal of these arks is to repatriate the animals once suitable habitat is restored, a causative agent of the decline (e.g. disease) is no longer a threat, or simply to supplement a fragmented population with additional brood stock. Thus, the near-term objective for these amphibian assurance colonies is to keep the animals alive long enough so that founder populations can reproduce and be sustainable. While there are certainly numerous breeding success stories, there are also several programs that require exogenous hormone stimulation of individuals for reproduction (e.g. *Anaxyrus baxteri, Anaxyrus boreas boreas*, and *Peltophryne lemur*) and may require assisted reproductive technologies (ART) such as *in-vitro* fertilization (IVF) for their continued propagation (e.g. *Rana sevosa*) [1]. For example, the endangered Wyoming toad (*Anaxyrus baxteri*) has only reproduced naturally twice in captivity and at least one gender has always received hormone therapy; yet, nearly 20,000 tadpoles were produced for release in 2009 and more than 100,000 for the program over a ten year period (personal communication, Bruce Foster SSP coordinator). Unfortunately, for many captive assurance species there is a growing need for ART because of the lack of knowledge about their ecology, especially the environmental and social cues that stimulate reproduction and how nutrition correlates to reproductive fitness.

Exogenous hormones have been used to stimulate amphibian breeding for more than 50 years [1-3]. The two hormones commonly employed for stimulating spermiation and ovulation in live animals are luteinizing hormone releasing hormone (LHRH) analog and human chorionic gonadotropin (hCG) with their effects extensively reviewed by several investigators [1,2,4-6]. Whitaker [5] provides a detailed review of trial and error hormone concentrations selected for assisted breeding in 16 different anuran species. His summary clearly revealed that the range of hormone concentrations used, most of them empirically chosen, appeared to be species-specific but were often passed down by habit or tradition (about 33% were not published studies). Similarly, Goncharov et al. [4] stimulated spawning in 37 different anuran species, using primarily LHRH but also hCG, and showed that successful gamete production for each species varied by the dosage (2 μg/kg up to 8 mg/kg), the number of treatments and the injection interval. In addition to the concentration of hormone appearing to vary widely between species, the response time and duration of gamete production following hormone administration has rarely been studied in detail for any species.

Although several good reviews exist describing how hormone administration for assisted breeding is species-specific [1,2,4-6], very few publications are available showing how sperm production and quality or the animals’ reproductive behavior are affected with variation in hormone concentration. This information is critical to captive breeding programs within zoos or aquariums that are regularly employing exogenous hormones for breeding as many pairings often fail to elicit amplexus behavior, exhibit poor egg fertilization rates, or animals release gametes in the absence of the other gender. For the most part, zoos and aquariums have typically used LHRH for assisted natural breeding, while academic studies employ hCG for collecting gametes to study fertilization and early embryonic development. The disparity between these two different approaches by institutional type may be related to their specific objectives. Whereas most university studies are using hormones as a tool to collect gametes for assisted fertilization or IVF, zoos are attempting to use the same compounds for assisted semi-natural breeding. The captive husbandry guidelines for *Anaxyrus baxteri, Peltophryne lemur*, and *Anaxyrus boreas boreas* all recommend LHRH concentrations in the range of 0.1-0.3 μg/g body weight. Having a better understanding of whether these programs are indeed using the optimal hormone concentration or correct hormone combination for breeding may improve the poor fertilization rates these programs frequently experience. The present study was conducted on the common American toad (*Anaxyrus americanus*), as a model species, for determining baseline hormone concentrations and potential toxicity issues before testing on endangered species. Obringer et al. [7] tested three different concentrations of LHRH on sperm production in *Anaxyrus americanus* and found a dose-dependent effect; however, a direct comparison of LHRH with hCG was not evaluated and sperm concentration was low compared to what we have found using hCG on another common species, *Anaxyrus fowleri*[1].

The objectives of this study were to compare the efficacy of four different concentrations of LHRH and hCG on sperm production over time. We measured the number of male *Anaxyrus americanus* that respond by producing spermic urine, and evaluated the quantity and quality of the semen by measuring the concentration, motility and forward progression of the spermatozoa. Once the best hormone concentrations from our treatment groups were determined, we then evaluated their effect on inducing reproductive behavior over time as determined by the number of animals in amplexus with females. The results from this study may have direct bearing on establishing more successful breeding protocols for numerous endangered species that a global community of zoos and aquariums are working with including *Rana sevosa, Anaxyrus boreas boreas, Anaxyrus baxteri*, *Atelopus zeteki*, and *Peltophryne lemur*.

## Methods

### Animals

Adult male American toads (*Anaxyrus americanus*) were collected from Northern Kentucky during the breeding season between May and June. The presence of calling, nuptial pads and pigmented vocal sacs indicated adult reproductive status. The toads were housed in groups of four in covered plastic enclosures (30.48 H x 38.1 W x 55.88 L cm) maintained at 26-28°C with water bowls and substrate. Animals were fed crickets three times a week that had been dusted with Reptical® and vitamin D supplementation. Males were selected of similar weight for the study with an average of 44.3 ± 2.4 g. Experiments were conducted in the lab from September-March, outside of normal breeding season to avoid any additional influence of seasonality on the effect of the hormones on spermiation. The project was approved by the Memphis Zoo’s IACUC (#001-11-01).

### Sperm collection and analysis

Spermic urine was collected from individual male toads following hormone administration treatments by holding the animal over a 150 mm Petri dish and gently spreading the hind legs apart with thumb and index finger which typically resulted in spermiation within 1–3 min as previously described [1]. Spermic urine was immediately placed into a 1.5 ml eppendorf tube, the volume measured and the sperm evaluated by placing 10 μl on a slide and viewed at 400x magnification using an Olympus CX41 phase-contrast microscope. Variables measured included number of males producing sperm, percent motility, concentration and quality of forward progression (FP). Forward progression was based on a qualitative scale of 0 to 5, where 0 = no movement, and 5 = rapid forward movement. Spermatozoa exhibiting beating flagella were considered motile, even if no FP was observed. This qualitative scale is commonly used to assess the quality of sperm progressive motility in mammals [8] as well as amphibians [9,10] and is used to develop a sperm motility index (SMI) as previously described [8]. The SMI was calculated as follows [% individual motility + (quality of motility x 20)] x 0.5. Sperm concentration was evaluated using a Neubaeur hemacytometer. In brief, 10 μl of sperm was mixed with 90 μl of 0.9% saline to inhibit sperm motility for counting; 10 μl of diluted sperm was placed in each chamber and the four corner squares of the grid counted for sperm. The average count for the two sides of the hemacytometer were then multiplied by the dilution factor and the conversion factor of 2500 and concentration expressed as number/ml.

### Study 1: Evaluation of hCG administration on sperm collection

Study 1 examined the dose-dependent effects of hCG administration on sperm production and motility characteristics over time. Lyophilized hCG (Sigma-Aldrich Co.; 2500 IU; C1063) was rehydrated using various amounts of sterile saline such that 100 μl contained 50, 100, 200, or 300 IU of hormone (expressed on an average body weight (BW) basis the treatments would be 1.13, 2.28, 4.51 and 6.77 IU/g BW). Following intra-peritoneal injection, animals were housed in plastic containers with aged tap water, approximately 2–3 cm deep, such that their abdomen was immersed to promote urine formation. Urine was collected from live American toads (n = 16/treatment) immediately after injection to confirm the absence of sperm and was subsequently collected at 3, 5, 7, 9, 12 and 24 h after injection and evaluated for the presence of spermatozoa, as previously described [2,7,10]. In some cases animals did not give a urine sample, thus the presence of sperm was not determinable and the percentage of responders was adjusted based on the number of urine collections obtained.

### Study 2: Evaluation of LHRH administration on sperm collection

For study 2 we evaluated the dose-dependent effects of LHRH administration on sperm production and motility characteristics over time. Lyophilized LHRH (Sigma-Aldrich Co.; 1 mg; L4513) was rehydrated using various amounts of sterile saline such that 20 μl contained 0.1, 1.0, 4.0, or 32 μg of hormone (expressed on an average BW basis the treatments would be 0.002, 0.022, 0.090, and 0.722 μg/g BW). The LHRH chosen for this study is a synthetic analog ([des-Gly10, D-Ala6]-LHRH ethylamide acetate) and is the standard LHRH used in many studies to date for inducing spermiation in amphibians as well as captive breeding programs worldwide. Following intra-peritoneal injection, animals were housed in plastic containers with aged tap water, approximately 2–3 cm deep, such that their abdomen was immersed to promote urine formation. Similar to Study 1, urine was collected from live American toads (n = 20/treatment) at 0, 3, 5, 7, 9, 12 and 24 h after injection and evaluated for the presence of spermatozoa, as previously described. There are four additional animals per treatment group in study 2 compared to study 1, as study 2 was based on an earlier pilot study conducted for power analysis, which included these four additional animals.

### Study 3: Effects of hCG and LHRH on amplexus behavior

Study 3 was designed to evaluate how the two hormones, hCG and LHRH, affect reproductive behavior by counting the number of males in amplexus (grasping of the female by the male) at the same time points that sperm production and quality had been evaluated in Studies 1 and 2. We evaluated amplexus behavior in toads receiving either 300 IU hCG (n = 12/trt) or 4 μg LHRH (n = 11/trt), compared to control (n = 12/trt) in which animals received 100 μl of sterile saline only. Hormone concentrations were determined in Studies 1 and 2, from which we chose the best concentration based on number of responders and sperm quantity. Following hormone administration, each male was paired with a single female *Anaxyrus americanus* in a small plastic shoebox enclosure (34.3 cm x 20.3 cm x 12.7 cm) assuring visual and physical contact throughout the duration of the experiment, and the number of animals in amplexus recorded for each time point by visual inspection at 0, 3, 5, 7, 9, 12 and 24 hrs for the three treatments. Females were not treated with hormones.

### Statistical analysis

Data were analyzed using the Generalized Estimating Equations (GEE) incorporating repeated measures over time and including the main effects of treatment and time, and the treatment by time interaction. We used GEE to accommodate for correlated longitudinal data over time using the linear model, which could also account for missing samples at specific time points due to the lack of urine collection at those time points. For the GEE regression parameters we report the Wald Chi-square as W. For binary data (sperm production and amplexus in response to hormones) we used the binary logistic model within GEE. Percent motility data were arcsine transformed prior to analysis. Differences between treatment means were evaluated by Fisher’s Protected Least Significant Difference test. All tests were two-tailed. Only those animals producing sperm were used for further treatment comparisons within concentration, motility, FP and sperm motility index (SMI). Values are expressed as Mean ± SEM. We considered differences significant when *P* < 0.05 and trending towards significance when *P* was between 0.05 and 0.10. All statistic analyses were performed using spss PASW 18 for Windows.

## Results

### Study 1: Effects of hCG on sperm characteristics

**Figure 1:**
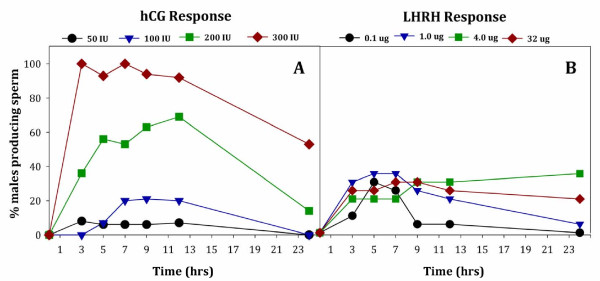
**Percentage of male *Anaxyrus americanus *producing sperm over 24 hrs after hormone administration with either hCG (Panel A; n = 16/trt) or LHRH (Panel B; n = 20/trt).** Male toads were treated with four different concentrations of hCG at 50 IU (circles); 100 IU (triangles); 200 IU (squares) or 300 IU (diamonds). Alternatively, males were treated with four different concentrations of LHRH at 0.1 μg (circles); 1.0 μg (triangles); 4 μg (squares) or 32 μg (diamonds).

Male *Anaxyrus americanus* administered with four different concentrations of hCG produced sperm in a dose-dependent manner, with 100% of the animals producing sperm when given 300 IU hCG (Figure 1A). All of the toads receiving 300 IU hCG were producing sperm at the first collection time point, 3 hrs post-administration of hormone. In comparison, the percentage of toads producing sperm at 3 hrs with 200 IU (36%), 100 IU (0%) or 50 IU hCG (8%) was much less (Figure 1A). No animals in the control group, receiving sterile saline only, produced sperm. We found a significant difference in the number of male toads producing sperm depending on the hCG concentration (W_3_ = 161.38, *P* < 0.0005) with the number of males producing sperm being highest after administrating 300 IU hCG (*P* < 0.0005 for all pair-wise comparisons between 300 IU hCG and any other concentration). Only one toad given 50 IU hCG produced sperm; thus, we did not include the 50 IU hCG concentrations in the analyses for sperm characteristics (motility, forward progression or concentration). The number of male toads producing sperm did not significantly vary from 3 to 12 hrs (*P* > 0.05), but decreased at 24 hours (*P* < 0.0005). At 24 hours post-administration of hormone, none of the toads treated with 50 or 100 IU hCG were releasing sperm and the number of toads having received 200 IU hCG declined in sperm production from 69% to 14% between 12 and 24 hrs, respectively (Figure 1A). Yet, 53% of the toads administered 300 IU hCG were still producing sperm 24 hrs post-administration.

**Figure 2:**
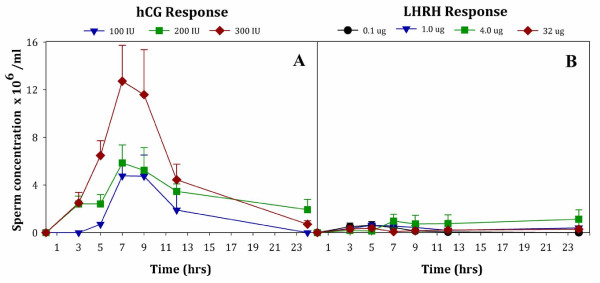
**Sperm concentration (x 10**^**6**^**/ml) produced by male *Anaxyrus americanus *over 24 hrs after hormone administration with either hCG (Panel A) or LHRH (Panel B).** Male toads were treated with 3 different concentrations of hCG at 100 IU (triangles); 200 IU (squares) or 300 IU (diamonds). Alternatively, males were treated with 4 different concentrations of LHRH at 0.1 μg (circles); 1.0 μg (triangles); 4 μg (squares) or 32 μg (diamonds). Only one male administered 50 IU hCG produced sperm and was not included in Panel A. Data are expressed as Mean ± SEM; lower SEM bars not shown. For number of animals comprising each treatment Mean see Figure 1.

Sperm concentration of male *Anaxyrus americanus* was highest after administering 300 IU hCG (Figure 2A; W_2_ = 8, *P* = 0.018) compared to other hormone regimens. Furthermore, the concentration varied at different times after hormone administration (W_6_ = 101.48, *P* < 0.0005). For example, after administering 300 IU hCG, sperm concentration was significantly higher (*P* < 0.05) at 7 and 9 hrs than at any other time points tested. There was a treatment by time interaction (W_10_ = 59.73, *P* < 0.0005), indicating that the magnitude and direction of the response to the hormone levels varied over the collection periods.

**Figure 3:**
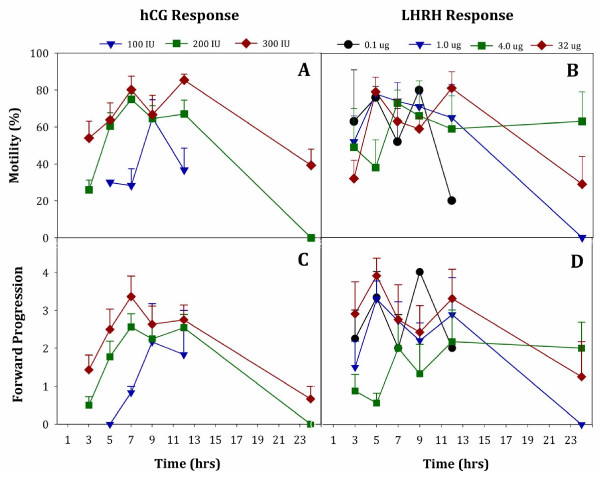
**Percent sperm motility from male *Anaxyrus americanus *treated with either hCG (Panel A) or LHRH (Panel B) over time after administration with either: 100 (triangles), 200 (squares), or 300 (diamonds) IU hCG compared to 0.1 (circles), 1.0 (triangles), 4 (squares) or 32 (diamonds) μg LHRH.** Forward Progression for these same sperm samples are shown in Panel **C** for hCG or Panel **D** for LHRH. Only one male American toad administered 50 IU hCG produced sperm for quality assessment and was not included in Panel A and C. Data are expressed as Mean ± SEM; lower SEM bars not shown. For number of animals comprising each treatment Mean see Figure 1.

Figure 3A shows the sperm motility after administration of 100–300 IU hCG. Sperm motility differed depending on the hCG concentration (W_2_ = 12.98, *P* = 0.002) and the time after hormone administration (W_5_ = 76.65, *P* < 0.0005). The treatment by time interaction for motility trended towards significance (W_8_ = 13.76, *P* = 0.09). Pair-wise comparisons of motility data differed significantly between the 300 IU hCG and lower concentrations of hCG (100 and 200 IU; *P* < 0.01 for both comparisons). Furthermore, the quality of FP sperm motility for male American toads differed among the 100, 200, and 300 IU concentrations (W_2_ = 8.41, *P* = 0.015). Sperm FP motility was highest when administering 300 IU hCG (Figure 3C), with the 200 IU and 300 IU concentrations almost differing statistically (*P* = 0.053), yet both of these concentrations differed from the 100 IU concentration (*P* < 0.05). Forward progressive sperm motility was highest between 5 hrs and 12 hrs after hormone administration (Figure 3C; W_5_ = 102.68, *P* < 0.0005). Similar to motility, the treatment by time interaction for FP trended towards significance (W_8_ = 14.46, *P* = 0.07).

### Study 2: Effects of LHRH on sperm characteristics

We found significant differences in the number of male toads producing sperm depending on the LHRH concentrations tested, 0.1-32 μg/per animal (Figure 1B; W_3_ = 36.6, *P* < 0.0005). Specifically, the number of male toads producing sperm differed between the lowest LHRH concentration (0.1 μg) and all the higher concentrations (*P* < 0.05) for the majority of time points where samples were collected. When compared to 300 IU hCG, all four concentrations of LHRH tested had low numbers of animals producing sperm at the first 3-hour time point; 0.1 μg (10%), 1.0 μg (30%), 4.0 μg (20%) and 32 μg (30%). While 300 IU of hCG induced 100% of the animals to produce sperm, the best response to LHRH had a maximum of only 35% of the animals producing sperm (Figure 1B). There was an effect of time on sperm production (W_5_ = 470.42, *P* < 0.0005), and a treatment by time interaction (W_14_ = 5982.61, *P* > 0.0005) indicating that the number of responders producing sperm changed over time. Sperm concentration of male *Anaxyrus americanus* was not significantly different between any of the four LHRH concentrations (W_3_ = 4.72, *P* = 0.19; Figure 2B). Sperm concentration was also similar between 3 hrs and 24 hrs after hormone administration (*P* > 0.05 for all pair-wise comparisons).

Figure 3B shows that sperm motility was not dependent on the dose of LHRH tested (W_3_ = 1.3, *P* = 0.73;). However, there were significant differences in sperm motility between different times after hormone administration (W_5_ = 31.4, *P* < 0.0005). Sperm motility differed between 3 hrs and 9 hrs (*P* = 0.012), was similar between 5 hrs and 12 hrs (*P* > 0.05 for all pair-wise comparisons), and decreased between 12 hrs and 24 hrs (*P* = 0.038). In addition, a treatment by time interaction was detected for sperm motility (W_14_ = 21733, *P* < 0.0005). Figure 3D shows the results of LHRH hormone administration on sperm Forward Progression. Forward progressive sperm motility of male *Anaxyrus americanus* was significantly different depending on the concentration of LHRH (W_3_ = 11.31, *P* = 0.01). Specifically, forward progression differed between dosages of 4 μg LHRH and either 0.1 μg LHRH or 32 μg LHRH (*P* < 0.05 for the two pair-wise comparisons) but not between 4 μg LHRH and 1 μg LHRH (*P* = 0.15). There were significant differences between different times after hormone administration (W_5_ = 28.99, *P* < 0.0005), as well as a treatment by time interaction (W_14_ = 139.38, *P* < 0.0005). Specifically, forward progression increased from 3 hrs to 5 hrs (*P* = 0.016), remained similar between 5 hrs and 12 hrs (*P* > 0.05) and then decreased between 12 hrs and 24 hrs (*P* = 0.001).

### Comparison between 300 IU hCG and 4 μg LHRH

**Figure 4:**
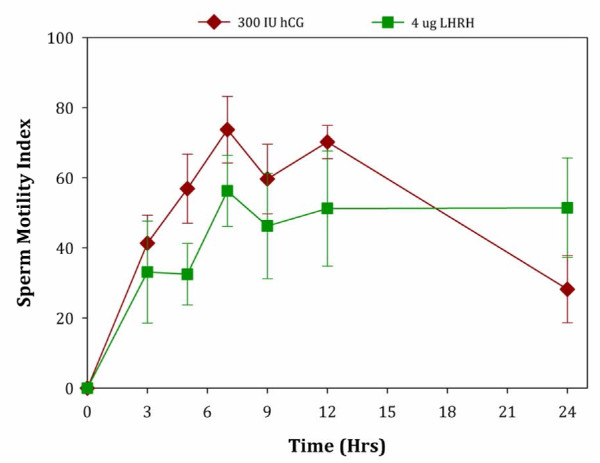
**A comparison of the sperm motility index (SMI) for *Anaxyrus americanus *treated with either 300 IU hCG (diamonds) or 4 μg LHRH (squares).** Data are expressed as Mean ± SEM. For number of animals comprising each treatment Mean see Figure 1.

Next, we chose the best hormone concentrations from each treatment group (hCG or LHRH) to compare sperm characteristics. While 300 IU hCG was clearly the best concentration in our experimental design for the first treatment group, the LHRH results were less clear. A concentration of 4 μg LHRH was chosen for direct comparison as this hormone concentration is closest to the level commonly used in breeding programs (for 25–40 g/body weight toads) and there was no difference in the number of animals responding, concentration, or motility between 4 μg and the 1 μg or 32 μg LHRH treatments. We found that male toads administered hCG had a greater number of responders (W_1_ = 63.15, *P* < 0.0005), and much higher sperm concentration (W_1_ = 18.6, *P* < 0.0005) than those males receiving LHRH. Average sperm concentration was 12 times higher in spermic urine collected from males receiving 300 IU hCG (12 x 10^6^/ml) compared to those receiving 4 μg LHRH (1 x 10^6^/ml). In contrast, these two treatments did not result in significant differences in sperm motility (W_1_ = 0.35, *P* = 0.55) or the quality of forward progressive motility (W_1_ = 3.63, *P* = 0.057). Similarly sperm motility index, which is an overall measure to evaluate the quality of sperm, did not differ between the hCG and LHRH concentrations evaluated (Figure 4; W_1_ = 2.54, *P* = 0.11).

### Study 3: Effects of hCG and LHRH on amplexus behavior

**Figure 5:**
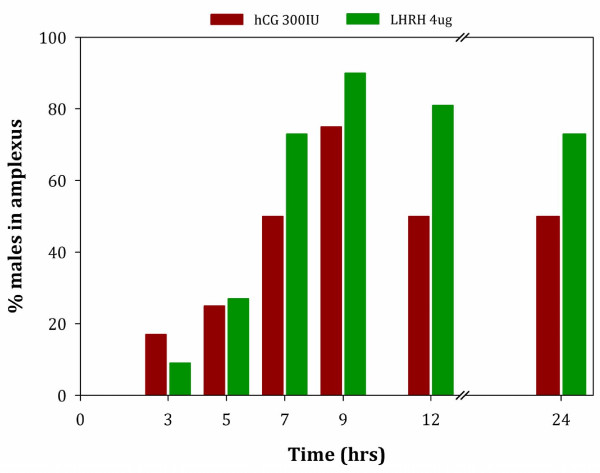
**The percentage of male *Anaxyrus americanus *amplexing females over time following hormone administration.** Males were treated with the optimal hormone concentrations from Study 1 and Study 2, specifically, 300 IU hCG (n = 12/trt) or 4.0 μg LHRH (n = 11/trt). Females were not treated with any hormones.

Figure 5 shows the reproductive behavioral response of male toads injected with either hCG (300 IU) or LHRH (4 μg). No male toads were observed in amplexus following administration of the control (saline only). The number of pairs amplexing was not significantly different between the control and either treatment at 3 hrs or 5 hrs post-hormone administration (Fisher's probability tests: *P* > 0.05 for all tests). In contrast, at 7 hrs, 9 hrs, 12 hrs, and 24 hrs, the number of pairs amplexing was higher when males were administered either 300 IU hCG or 4 μg LHRH compared to the control group (Fisher's probability tests: *P* ≤ 0.01 for all tests). When considering the overall interaction effect of treatment over time between the two treatments, hCG or LHRH, there was not a significant difference in the number of pairs’ amplexing for 300 IU hCG and 4 μg LHRH (GEE: W_1_ = 0.35, *P* = 0.35). However, the number of males that were observed in amplexus was significantly different at specific individual time points after treatment (W_4_ = 27.34, *P* < 0.0005). The number of toads observed in amplexus was lower at 3 hrs and 5 hrs post-hormone administration than at any later time (*P* < 0.0005 for all pair-wise comparisons). As shown in Figure 5, the number of toads observed in amplexus was highest at 9 hrs post-hormone administration (91% when treated with LHRH and 75% when treated with hCG). Although the number of toads observed in amplexus was still relatively high at 24 hrs (73% when treated with LHRH and 50% when treated with hCG), there was a significant drop between 9 hrs and 24 hrs (*P* = 0.015; Figure 5). No treatment by time interaction was detected (W_4_ = 2.83, *P* = 0.59).

## Discussion

Our research demonstrates that amphibian captive breeding programs that seek to utilize exogenous hormones for assisted reproduction need to carefully evaluate the optimal hormone concentration for their particular species. If the goal is assisted natural breeding, rather than *in vitro* fertilization, meticulous consideration must also be given to which hormones stimulate reproductive behaviors as well. In our study, the best hCG concentration tested, where 100% of the animals responded by producing sperm, was 300 IU. This same concentration of hCG has also been found to be extremely effective in stimulating spermiation in *Anaxyrus baxteri *[1,2,9], *Anaxyrus boreas boreas *[1], *Anaxyrus fowleri *[1], *Anaxyrus houstonensis* (personal communication, Paul Crump) and *Peltophryne lemur* (personal communication, Andrew Lentini). Although this concentration of hCG is extremely effective in stimulating male Bufonidae to produce spermic urine, species-specific differences can be found in the density of sperm produced. In our lab, we have found that seven Bufonidae species (in the 20–75 g range) produce spermic urine after hCG administration of 300 IU or higher; however, one Ranid species of similar weight (*Rana pipiens*) produced low quantities of sperm with often variable responses when treated with similar hCG concentrations [2]. This further supports our concept that developing a protocol in one taxonomic species may not extrapolate exactly to another, but that fine-tuning is necessary on hormone efficacy to optimize a breeding strategy. However, the basic principles learned about one taxa may provide a good starting point for more developed studies.

By testing various concentrations of hCG from 50–300 IU we were able to show a clear dose-dependent and dramatic increase in the number of male *Anaxyrus americanus* producing sperm, along with an increase in concentration of sperm. In contrast, LHRH did not show a clear dose-dependent effect on the sperm parameters measured. None of the LHRH treatments tested produced as high a concentration of sperm as shown with the 100–300 IU hCG treatments. Moreover, the number of animals producing sperm in response to LHRH (~35%) was much less than the 100% achieved with 300 IU hCG. Interestingly, the individual sperm characteristics, motility, forward progression, and SMI from the optimal LHRH treatment group were not different from the 300 IU hCG group. This suggests that hormone type and concentration can be altered to induce more animals to produce greater quantities of sperm, but that these hormones did not directly affect the movement patterns or quality of mature ejaculated spermatozoa. While it is possible that increasing the concentration of hCG above 300 IU/animal might increase sperm concentration or possibly the number of animals in amplexus, we chose to stop at a point where 100% of the animals were producing sperm of good quality that would lead to high rates of natural or artificial fertilization without compromising the health of the animals by over-dosing them. Furthermore, we recognize that our behavioral responses were not optimized (75% of the pairs in amplexus with hCG compared to 91% with LHRH) and that higher doses may have increased numbers of males exhibiting amplexus. Future studies will examine the effect that combinations of the two hormones might have on reproductive behaviors at these concentrations without increasing hormone levels to possibly dangerous concentrations. This method of cautious experimentation and our stopping point within our treatments is necessary considering we are outlining a hormonal strategy for developing such protocols in critically endangered toad species where losses to the assurance colonies could be catastrophic.

Very few studies have been conducted in male amphibians directly testing these two different hormones at four or more concentrations. Michael et al. [11] evaluated different concentrations of hCG and LHRH in female *Eluetherodactylus coqui* and, in contrast to our results with male toads, found that LHRH stimulated more female *coqui* to ovulate than the hCG concentrations they tested. The difference between these results and ours are likely due to species or gender differences in the response to these hormones. Our lab has found that toads within the family Bufonidae tend to respond in a similar fashion to hCG across species; yet, *Rana pipiens* seem to respond better to LHRH [2,12] and these results are often concentration dependent. Understanding the *a priori* hormone levels that stimulate optimal spermiation or ovulation is critical for long-term breeding programs where captive facilities may utilize these protocols for endangered or threatened species. Without this prior knowledge on how a species will respond to varying hormone concentrations (possibly spanning several orders of magnitude), investigators may be tempted to conclude that their species’ lack of ovulation or spermiation may limit hormone induction as a conservation tool. For example, Mann et al. [13] concluded that a suite of hormones tested, at a single concentration, did not work well in *Litoria raniformis*. However, a more robust level of hormone testing may have yielded a larger number of animals ovulating, and could be explored.

Recent studies on *Anaxyrus boreas boreas* suggest that higher LHRH or hCG concentrations, or even priming regimens, may be more effective than what was historically employed in the captive breeding and reintroduction program for this threatened species (Natalie Calatayud and Kevin Thompson, unpublished). Knowing that the optimal hormone concentration could be orders of magnitude higher than historically employed for inducing spermiation or ovulation in this species, raises the question of how to interpret other studies that are dependent upon hormone therapy to answer other physiological processes, such as the importance of hibernation in captive breeding [14]. Often times, how investigators arrived at optimal hormone levels is not clear and reports indicate that concentrations were: 1) chosen from previously published reports on other species; 2) determined in a pilot study; 3) established in a limited number of animals; 4) measured but the data was not shown; or 5) inconclusive leading to the need to go back and understand hormone efficacy due to less than hoped for results [15-17]. To be used as a conservation tool for the growing number of endangered amphibian species that will need reproductive intervention to prevent extinction, it is valuable that hormone efficacy trials be continued so that future studies on other species can be modeled likewise. Studies beginning with very low concentrations of hormone and working up to higher levels is imperative to strike a balance between using enough hormone to obtain gametes for reproduction while not administering too high a dose that will cause health problems or even possibly death of the animal.

Another important finding from our study was that the hormones’ effects on sperm concentration, motility and forward progression display a time-dependent trend within a treatment (more visibly obvious with hCG than LHRH). American toad sperm concentration peaks between 7–9 hrs post hCG administration at 12 x 10^6^ sperm/ml and declines steadily to about 4.5 x 10^6^ and 2 x 10^6^ sperm/ml at 12 and 24 hrs, respectively. However, sperm concentrations for our LHRH treatment were low compared to hCG and typically were below 1 x 10^6^ sperm/ml. This low sperm concentration following administration of 4.0 μg LHRH in our study is similar to levels reported by Obringer et al. [7] for *Anaxyrus americanus* administered 4.0 μg LHRH via subcutaneous injections. However, they report an average concentration of 4.9 x 10^6^ sperm/ml when providing the same hormone dose via intra-peritoneal injections. The range in sperm concentration for their study was 0.1 to 24 x 10^6^ sperm/ml, although 33% of their values were below 1 x 10^6^ sperm/ml indicating that one third of their data readings were comparable to our values when using the same hormone concentration and route of administration. The average sperm concentration value reported by Obringer et al. [7] are nearly five times higher than what we observed, due to a couple animals with very high sperm concentration. The discrepancy in our results from theirs with LHRH may be due to seasonal timing issues. Obringer et al. [7] initiated their study on newly captured animals during the breeding season with all experiments conducted shortly thereafter (April-June). In contrast, our animals had been held in captivity for more than a year prior to the start of the study, were not hibernated, and all experiments were conducted outside of the breeding season. For endangered toads held long-term in captivity as part of a breeding program, our study scenario is likely more realistic of the challenges confronted by using non-hibernated individuals without the appropriate environmental cues to stimulate natural reproduction (hence the need for hormones in the first place).

The time an amphibian takes to respond to a hormone treatment has direct bearing on how amphibian captive breeding programs time their series of hormone injections. Information from our studies highlights not only the importance of testing various hormone concentrations, but also the importance of carrying out a timed experimental sampling protocol such that the peak behavioral or physiological responses being measured are known. For example, studies in our lab with *Rana pipiens* discovered that sperm production following hormone administration occurred within 30 minutes and had stopped by 2 hours (unpublished data). If the same sampling period had been followed in the *Rana pipiens* experiments as with this study on *Anaxyrus americanus*, one might incorrectly assume that the hormone was ineffective, when the reality would be that the optimal time for sperm collection had been missed. Studies like these allow for protocol development on gamete synchronicity or the “timed release” of both sperm and eggs to maximize artificial fertilization or assisted natural breeding. After a review of several Bufonid captive breeding programs it became apparent that both male and female amphibians were receiving hormone stimulation simultaneously during the morning. In the case of the *Anaxyrus americanus*, this would result in an asynchronous release of gametes. Typically, female *Anaxyrus americanus* release eggs 12–24 hours post-hormone administration, but optimal sperm production in the male occurs 7–9 hours post-hormone administration and would be declining rapidly after 12 hours. These results indicate that the effect of hormone on spermiation has a limited duration. Several U.S. zoos working with the authors report that often females lay eggs following hormone stimulation but that poor fertilization was noted. This poor fertilization may be a result of the males already being in the refractory period and no longer producing sperm. Thus, an understanding of optimal or peak gamete release in relation to hormone administration assists in the development of a timed AI program for amphibians and how best to stagger the hormone injections for optimal fertilization and inception of reproductive behaviors.

While we were fairly confident that hCG was the optimal hormone for collecting sperm from *Anaxyrus americanus* to use for artificial fertilization experiments we needed to test whether replacing LHRH with hCG in several captive breeding programs for other Bufonids would affect their induced mating strategies. Several pilot studies were conducted at Central Park Zoo, Fort Worth Zoo and Sybille Wildlife Recovery Center in Wyoming to see whether amplexus or the percent of fertilized eggs increased in *Anaxyrus baxteri* or *Peltophryne lemur*. Every institution reported seeing fewer animals in amplexus; thus, fewer fertilized eggs, when administering hCG instead of LHRH. Hence, we went back and created a series of experiments to understand the impact these two hormones had on reproductive behavior. We found that over 91% of the pairs given LHRH were in amplexus 9 hrs post-administration, while 75% of the animals given hCG were in amplexus at the same time period. These two protein hormones have very different binding receptors and modes of action, yet both have non-gonadal targeted tissues with potential overlapping results on reproductive behaviors with varying degrees of responsiveness.

In the rat brain hippocampus, LHRH acts as a neurotransmitter linking actions inside the central nervous system where it facilitates reproductive behaviors to peripheral endocrine effects [18]. In addition, LH/hCG receptors have also been found in the rat hippocampus, impacting specific reproductive behaviors [19]. One of the hippocampus’ roles is chemical and hormonal sensing, whereby it exerts cognitive control over specific aspects of the hypothalamic-pituitary-adrenal axis impacting reproduction as well as memory cognition and arousal [20]. In some amphibian species, it is likely that similar actions are occurring and that both LHRH and LH/hCG homologues modify neuronal excitability within the hippocampus, thereby modulating hippocampal function and downstream behaviors, although at varying levels of stimulation. Androgen receptors are also located within the hippocampus [20] and steroid biosynthesis following exogenous hormone administration may be another indirect route for stimulating amphibian spermiation or onset of reproductive behaviors. Binding of hCG to receptors in the testis has been shown to initiate steroid production and spermiation in several amphibians including *Rana nigromaculata*[21], *Xenopus laevis*[22] and *Bufo marinus*[23]. Similarly, administration of LHRH has been shown in several amphibian species to increase androgen production, resulting in increased reproductive behaviors and spermiation [24,25]. Hence, both LHRH and hCG stimulate similar steroidogenic pathways although the effectiveness of each exogenous hormone on reproductive behaviors or spermiation may be species-, dosage-, or hormone-specific.

Our results suggest that although hCG may be optimal for collecting gametes for artificial fertilization, the use of this hormone alone is not sufficient for inducing breeding and overcoming reproductive behavioral challenges. Previous studies by our lab have used both hormones in a cocktail mixture to induce *Anaxyrus baxteri* to ovulate large numbers of eggs when given as a priming hormone [9]. Future studies will test whether combining the two hormones in a cocktail formula may provide higher fertilization rates by increasing the sperm concentration while not sacrificing the number of animals that can be induced to amplex. Although we were able to achieve a 91% amplexus rate with LHRH up to 9 hrs in this series of experiments with *Anaxyrus americanus*, many programs using these same hormones, sometimes at higher concentrations than we tested, still report low numbers of animals in amplexus.

## Conclusions

In summary, research on appropriate hormone types and concentrations can be beneficial to captive breeding programs for stimulating optimal sperm and egg production in amphibians. In addition, the hormone of choice may depend upon whether the purpose is to collect gametes for artificial fertilization or to assist natural breeding and amplexus. Regardless of the hormone chosen, understanding when the peak and refractory period occurs for gamete production will likely dictate the timing of ‘when’ and ‘if’ successful reproduction takes place. These protocols should be developed early in a research program such that future studies on physiological or behavioral control mechanisms are not impacted by inappropriate hormone regimens. This is especially important for reintroduction programs of endangered species where producing brood stock is a key component for recovery. Another priority for amphibian conservation programs should be the development of techniques for shipping sperm between institutions for artificial fertilization, hence allowing for greater maintenance of genetic diversity and reducing stress from transporting animals. Appropriate hormone protocols for targeted species will be the key to the success of any particular conservation effort. Lastly, more research is needed to understand how often animals can be administered hormones to stimulate sperm production or ovulation and whether the exogenous hormones affect fertilization rates, tadpole development, metamorphosis and/or production of subsequent generations.

## Competing interests

The authors declare they have no competing interests.

## Authors’ contributions

AK designed the study, funded the project, helped with analysis of the data, and wrote the manuscript. JB helped analyze the data, conducted all statistics and helped with the manuscript. CV helped design the project, conducted the work, made all the Figures and edited the manuscript. CM and MC conducted the experiments. All authors read and approved the final manuscript.

## References

[B1] Kouba AJ Vance CK Applied reproductive technologies and genetic resource banking for amphibian conservation Reprod Fertil Dev 2009 21 719 737 10.1071/RD09038 19567216

[B2] Kouba AJ Vance CK Willis EL Artificial fertilization for amphibian conservation: Current knowledge and future considerations Theriogenology 2009 71 214 227 10.1016/j.theriogenology.2008.09.055 19026442

[B3] Wiltberger PB Miller DF The male frog, *Rana pipiens*, as a new test animal for early pregnancy Science 1948 107 198 17791766 10.1126/science.107.2773.198

[B4] Goncharov BF Shubravy OI Serbinova IA Uteshev VK The USSR programme for breeding amphibians, including rare and endangered species Int Zoo Yearb 1989 28 10 21

[B5] Whitaker BR Wright KM, Whitaker BR Reproduction Amphibian Medicine and Captive Husbandry 2001 Malabar, Florida: Krieger Publishing Co 285 299

[B6] Roth TL Obringer AR Holt WV, Pickard AR, Rodger JC, Wildt DE Reproductive Science and Integrated Conservation Reproductive research and the worldwide amphibian extinction crisis 2003 Cambridge: Cambridge University Press 359 374

[B7] Obringer AR O'Brien JK Saunders RL Yamamoto K Kikuyama S Roth TL Characterization of the spermiation response, luteinizing hormone release and sperm quality in the American toad (*Bufo americanus*) and the endangered Wyoming toad (*Bufo baxteri*) Reprod Fertil Dev 2000 12 51 58 10.1071/RD00056 11194557

[B8] Garde JJ Soler AJ Cassinello J Crespo C Malo AF Espeso G Gomendio M Roldan ER Sperm cryopreservation in three species of endangered gazelles (*Gazella cuvieri, G. dama mhorr, and G. dorcas neglecta*) Biol Reprod 2003 69 602 611 10.1095/biolreprod.102.012914 12700201

[B9] Browne RK Seratt J Vance C Kouba A Hormonal priming, induction of ovulation and in-vitro fertilization of the endangered Wyoming toad (*Bufo baxteri*) Reprod Biol Endocrinol 2006 4 34 10.1186/1477-7827-4-34 16790071 PMC1524778

[B10] Kouba AJ Vance CK Frommeyer MA Roth TL Structural and functional aspects of *Bufo americanus spermatozoa*: effects of inactivation and reactivation J Exp Zool A Comp Exp Biol 2003 295 172 182 12541301 10.1002/jez.a.10192

[B11] Michael SF Buckley C Toro E Estrada AR Vincent S Induced ovulation and egg deposition in the direct developing anuran *Eleutherodactylus coqui* Reprod Biol Endocrinol 2004 2 6 10.1186/1477-7827-2-6 14748925 PMC340388

[B12] Licht P Gibbons EF Jr, Durrant BS, Demarest J Reproductive Physiology of Reptiles and Amphibians Conservation of endangered species in captivity: An interdisciplinary approach 1995 New York, NY USA: State University of New York Press 169 186

[B13] Mann RM Hyne RV Choung CB Hormonal induction of spermiation, courting behavior and spawning in the southern bell frog, *Litoria raniformis* Zoo Biol 2010 29 774 782 10.1002/zoo.20331 20549714

[B14] Roth TL Szymanski DC Keyster ED Effects of age, weight, hormones, and hibernation on breeding success in boreal toads (*Bufo boreas boreas*) Theriogenology 2010 73 501 511 10.1016/j.theriogenology.2009.09.033 20004010

[B15] Trudeau VL Somoza GM Natale GS Pauli B Wignall J Jackman P Doe K Schueler FW Hormonal induction of spawning in 4 species of frogs by coinjection with a gonadotropin-releasing hormone agonist and a dopamine antagonist Reprod Biol Endocrinol 2010 8 36 10.1186/1477-7827-8-36 20398399 PMC2873446

[B16] Mansour N Lahnsteiner F Patzner RA Motility and cryopreservation of spermatozoa of European common frog, *Rana temporaria* Theriogenology 2010 74 724 732 10.1016/j.theriogenology.2010.03.025 20537698

[B17] Byrne PG Silla AJ Hormonal induction of gamete release, and in-vitro fertilisation, in the critically endangered Southern Corroboree Frog Pseudophryne corroboree Reprod Biol Endocrinol 2010 8 144 10.1186/1477-7827-8-144 21114857 PMC3014959

[B18] Jennes L Eyigor O Janovick JA Conn PM Brain gonadotropin releasing hormone receptors: localization and regulation Recent Prog Horm Res 1997 52 475 491 9238864

[B19] Lei ZM Rao CV Neural actions of luteinizing hormone and human chorionic gonadotropin Semin Reprod Med 2001 19 103 109 10.1055/s-2001-13917 11394198

[B20] Lathe R Hormones and the hippocampus J Endocrinol 2001 169 205 231 10.1677/joe.0.1690205 11312139

[B21] Kobayashi T Sakai N Adachi S Asahina K Iwasawa H Nagahama Y 17 alpha,20 alpha-Dihydroxy-4-pregnen-3-one is the naturally occurring spermiation-inducing hormone in the testis of a frog, *Rana nigromaculata* Endocrinology 1993 133 321 327 10.1210/en.133.1.321 8319579

[B22] Kelley DB Pfaff DW Hormone effects on male sex behavior in adult South African clawed frogs, *Xenopus laevis* Horm Behav 1976 7 159 182 10.1016/0018-506X(76)90045-3 955579

[B23] Iimori E D'Occhio MJ Lisle AT Johnston SD Testosterone secretion and pharmacological spermatozoal recovery in the cane toad (*Bufo marinus*) Anim Reprod Sci 2005 90 163 173 10.1016/j.anireprosci.2005.01.010 16257605

[B24] Zoeller RT Moore FL Seasonal changes in luteinizing hormone-releasing hormone concentrations in microdissected brain regions of male rough-skinned newts (*Taricha granulosa*) Gen Comp Endocrinol 1985 58 222 230 10.1016/0016-6480(85)90338-7 3888777

[B25] Moore FL Boyd SK Kelley DB Historical perspective: Hormonal regulation of behaviors in amphibians Horm Behav 2005 48 373 383 10.1016/j.yhbeh.2005.05.011 15992801

